# The effect of oncology-specific suicide prevention program on oncology nurses’ levels of suicide literacy, suicide stigma, and efficacy perception for suicide risk management: a randomized controlled study

**DOI:** 10.1007/s00520-026-10854-0

**Published:** 2026-06-08

**Authors:** Sevda Öztürk, Duygu Hiçdurmaz

**Affiliations:** Department of Psychiatric Nursing, Hacettepe University Faculty of Nursing, Adnan Saygun Street, Block D, Flat 1, Samanpazarı, Ankara, PO 06100 Turkey

**Keywords:** Cancer, Oncology nursing, Psycho‐oncology, Suicide, Suicide awareness, Suicide prevention

## Abstract

**Objective:**

Cancer patients have a higher suicide risk than the general population. Oncology nurses play a critical role in assessing and managing this risk. However, they often lack the necessary awareness, knowledge, and skills. This study developed an Oncology-Specific Suicide Prevention Program (OSP) for oncology nurses. It aims to assess how the OSP affects nurses’ suicide literacy, suicide stigma, and efficacy in suicide risk management.

**Method:**

Researchers developed the OSP based on findings from cancer-related suicide studies. The intervention included three structured online sessions for the experimental group, each session addressing specific aspects of suicide prevention strategies in oncology care. Researchers conducted a randomized controlled trial with 86 nurses from two oncology hospitals in Ankara. Nurses were randomly assigned to an experimental group (*n* = 43) or a control group (*n* = 43) after stratifying by years of work experience. The control group received usual in-service training. Researchers collected data using the descriptive information form, the literacy of suicide scale, the stigma of suicide scale, and the efficacy perception scale for suicide risk management for oncology nurses.

**Results:**

The experimental group showed a significant increase in suicide literacy (*p* < 0.001) and efficacy perception for suicide risk management (*p* < 0.001). They also showed a decrease in suicide stigma (*p* = 0.013) compared to the control group. These effects were measured at the end of the program and 3 months later.

**Conclusion:**

Integrating the OSP into in-service training for oncology units and national and international suicide prevention strategies is suggested.

**Supplementary Information:**

The online version contains supplementary material available at 10.1007/s00520-026-10854-0.

## Introduction

Cancer is a disease that causes physical, emotional, psychological, and economic challenges to individuals, their families, and communities. Many challenges experienced by cancer patients during the cancer treatment process, such as treatment side effects [1, 2], high symptom burden [3], low quality of life, lack of social support [4], and being in a socioeconomically disadvantaged environment [5], make them more vulnerable to suicide risk. The suicide mortality rate for cancer patients is about 2–5 times higher than the expected rate in the general population [3, 6, 7]. A meta-analysis study conducted in the last 20 years by Du et al. (2020) showed that the mortality rate due to suicide in cancer patients was 39.72/100,000 per year [8], while the World Health Organization stated that global suicide rate was 9.2/100,000 in 2019 [9]. These findings emphasize the critical need to develop effective suicide prevention strategies within the field of oncology in response to the alarming suicide risk among cancer patients.

Healthcare professionals play a crucial role in recognizing and managing suicide risk in cancer patients because they are considerably proximal to the patients. Studies have reported that individuals who attempted suicide had contact with healthcare professionals very recently before their attempted suicide [10, 11]. These results emphasize the importance of oncology healthcare professionals identifying suicide risks in cancer patients before discharge and connecting them with appropriate resources. Oncology nurses possess care experience, disease-specific knowledge, and close physical and psychological proximity to patients, which positions them as key contributors to the recognition and management of suicide risk. However, previous studies show that oncology nurses are unaware of the suicide risk of cancer patients [12], while those who are aware lack specific knowledge and skills in managing suicide risk, such as risk assessment tools and effective communication strategies [12–14]. The stigmas specific to oncology about suicide make it more complicated to recognize the suicide risk in this field and also hinder awareness [13]. In addition, uncertainty about roles [13, 14] and disagreements among team members often cause challenges for nurses in coping with this risk and strengthen the perception of professional incompetence of oncology nurses [13].

Previous literature lacks a comprehensive study on the development of awareness in oncology nurses to recognize and prevent the risk of suicide in cancer patients. The literature emphasizes that current suicide prevention programs lack approaches specific to the field-of-study target group in suicide prevention programs to enhance their effectiveness [15, 16]. Therefore, it is essential to implement an awareness and skill development program for oncology nurses. Thus, the present study developed an Oncology-Specific Suicide Prevention Program for Oncology Nurses (OSP) to address the needs for suicide prevention in oncology. This program addresses the specific risk factors associated with the oncology population and the psychosocial challenges commonly faced by both nurses and patients. It also includes appropriate sources of support based on the structure of psychosocial services specific to our country. Current suicide prevention programs’ duration varies widely, ranging from as brief as 45 min to as extensive as 2 days [17]. The OSP offers a comprehensive approach by incorporating oncology-specific requirements and role-plays for suicide risk management. It consists of three sessions, each lasting 2 h, and is thoughtfully designed for online delivery to ensure accessibility and effectiveness. This online structure was chosen to facilitate better time management for practicing nurses. Research has shown that web-based suicide prevention programs are effective at improving knowledge, self-efficacy, and prevention behaviors [18, 19] and that face-to-face programs are not superior to online approaches [20, 21].

The OSP may help to improve the awareness, knowledge, and skills of nurses in identifying and managing suicide, making them more effective and fostering a positive change in their attitudes toward stigmatization. The primary purpose of this study was to determine the effect of the OSP on the nurses’ level of suicide literacy, suicide stigma, and efficacy perception for suicide risk management through a randomized controlled trial. A secondary aim was to determine the relations in oncology nurses’ reported outcomes regarding changes of suicide literacy, suicide stigma, and efficacy perception for suicide risk management. The hypothesis for this study is as follows:

At the end of the OSP and the 3-month follow-up, there are significant differences in the oncology nurses’ levels of suicide literacy, suicide stigma, and efficacy perception for suicide risk management when compared to the pre-program and control group.

There are significant relationships between the changes in the baseline-posttest and baseline-follow-up measurements of oncology nurses’ suicide literacy, suicide stigma, and efficacy perception for suicide risk management.

## Method

### Study design and settings

This study was a randomized controlled trial conducted to determine the effect of the OSP on the oncology nurses’ level of suicide literacy, suicide stigma, and efficacy perception for suicide risk management. The study was designed as a two‐arm study, with participants randomly allocated to the experimental group (the OSP) and the control group. Hospitals where we conducted this study followed the standard in-service training protocols mandated by the Ministry of Health. Thus, the control group continued with the standard professional development and in-service training protocol, while the intervention group received the OSP in addition to these routine programs. Furthermore, the control group was provided with written and verbal assurance from the research team that the OSP would be accessible upon request after data collection.

The study was conducted with nurses in two oncology hospitals, including inpatient clinics and outpatient clinics in the Ankara Metropolitan Municipality province.

### Participants

The G-Power software package calculated the sample size for the two groups and the three measurements using a type-1 error of 0.05 and a type-2 error of 0.20 (1 − *β* = 0.80 power level), with an effect size of 0.25. The calculated number of participants for the sample was 78 [22]. Considering a 10% dropout rate during the study, the total sample size was 86.

The inclusion criteria for the sample of the study were as follows:

- Volunteering to participate in the study
- Working as a nurse in oncology, including inpatient or outpatient clinics
- Demonstrating the ability to use a smart mobile phone or computer

The exclusion criteria from the study were as follows:

- Having training for suicide prevention
- Working in a pediatric oncology clinic
- Having experience of working in a psychiatric clinic or being a postgraduate psychiatric nursing student
- Failure to demonstrate the ability to use the online meeting application (Zoom) in the pre-intervention phase when the authors provided information and pilot try about the program
- Not attending two out of three sessions of the training program

Researcher 1 coordinated clinic visits with the guidance of the hospitals’ nurse managers to reach out to the participants. The visits were scheduled based on the nurses’ availability, and the nurses were invited to participate in the study. Out of the 112 eligible oncology nurses assessed for the study, 25 did not volunteer to participate, and one, a postgraduate student in psychiatric nursing, was excluded. Consequently, the study’s sample consisted of 86 nurses.

### Randomization

The researchers used the stratified randomization method to select groups of nurses based on their years of work experience. Previous studies have indicated that nurses with more work experience tend to have less negative attitudes toward suicide [23, 24]. The researchers categorized nurses’ work experience levels as follows: less than 1 year, 1–3 years, 4–9 years, 10–15 years, and more than 15 years to ensure similarity between the experimental and control groups based on established milestones for nurses’ occupational competence years [25, 26]. Before randomization, participants were assigned numbers, written on differently colored cards, and folded by stratum. A coin toss determined the starting stratum for randomization. A nurse unaffiliated with the research team made the selections, and the first choice was allocated to the experimental group. Consequently, 43 nurses were assigned to the experimental group and 43 to the control group.

### Intervention

The OSP is a suicide prevention program designed to develop awareness and enhance the skills of oncology nurses in identifying and managing suicide risks in cancer patients. The program aims to improve oncology nurses’ understanding of suicide and their ability to manage suicide risk, reducing suicide stigma and ensuring appropriate management of suicide risk in cancer patients. The program content is not just theoretical, but it integrates knowledge and skills developed following the research on suicide and suicide risk in oncology. The training program is designed to be interactive, using teaching techniques such as games, case analysis, role-play, experience sharing, question-and-answer, and discussion to ensure the permanence of the training knowledge and skills. Researchers 1 and 2 developed the content and structure of the group sessions. Before the study commenced, the developed OSP was reviewed by five experts, including three professors and two associate professors specializing in consultation-liaison psychiatry, clinical psychiatric nursing, and psychosocial care in oncology.

The program was finalized in accordance with their feedback. The OSP program consists of three sessions, each lasting 90–120 min.

#### Theoretical Framework of OSP

The OSP delivers evidence-based suicide prevention interventions specifically designed for oncology care. The OSP aims to improve oncology nurses’ understanding of suicide among cancer patients, support suicide risk assessment through therapeutic communication, and manage risk by coordinating psychosocial resources. Each session addresses a specific objective and provides guidance for practical implementation. The content of the OSP is grounded in the gatekeeper approach, a recognized suicide prevention intervention [27]. Gatekeeper training is designed to educate individuals who work with populations at elevated risk of suicide, equipping them to assess and refer at-risk individuals to appropriate services [17, 27, 28]. Nurses are identified as a “selected or assigned group” for gatekeeper training due to their professional expertise [27]. Although the content of gatekeeper training may vary according to the professional role of the gatekeeper, the specific suicide prevention needs [17, 28], the characteristics of the target group, and the context in which the intervention occurs, the core components of preparing, connecting, understanding, assisting, and networking are consistently included in the gatekeeper training model [27]. In this study, the three OSP sessions are organized around these five core components (preparing, connecting, understanding, assisting, and networking). In this framework, the first OSP session includes a “preparing” section that addresses norms, expectations, and the rationale for suicide prevention within the target group, as well as a “connecting” section focused on understanding suicide and increasing awareness of attitudes toward suicide. The second session encompasses the “understanding” and “assisting” components, which provide knowledge and skills for recognizing suicide risk. The third session addresses the “networking” component, which emphasizes practicing and internalizing communication and suicide risk management skills, and coordinating psychosocial resources. Additionally, each session integrates specific suicide theories, risk management strategies from mental health guidelines, and evidence-based risk management approaches that are described in detail in the following paragraphs.

The initial session, “Introduction to Understanding Suicide,” aims to increase oncology nurses’ awareness of the elevated suicide risk among cancer patients and related psychosocial factors such as anxiety, pain, care burden, socioeconomic challenges, loss, grief, loneliness, guilt, hopelessness, and emotional distress. Joiner’s (2007) Interpersonal Theory of Suicide provides a framework for understanding these factors, explaining that suicidal tendencies stem from perceived burdensomeness, thwarted belongingness, and acquired capability [29, 30]. Integrating this theoretical perspective highlights how suicide stigma influences risk awareness and interpretation among healthcare professionals, supporting a more comprehensive understanding of suicide mechanisms in cancer patients.

After the initial session, the next session focuses on suicide risk assessment, following recommendations from the American Psychiatric Association [31]. The OSP draws on the perspective of the Collaborative Assessment and Management of Suicidality (CAMS) intervention, an evidence-based practice that emphasizes empathetic assessment [32]. This approach involves asking about patients’ suicidal thoughts, plans, or behaviors and exploring the underlying meaning and function of suicide, such as seeking relief from pain. Within the CAMS framework, clinicians work with patients through open communication to address life challenges related to suicidality and unmet needs, support practical coping strategies, and develop crisis plans [32]. To apply these principles, the session uses case studies, videos, and role-play, allowing oncology nurses to practice risk-assessment techniques. The session also identifies barriers to the risk-assessment process and offers strategies to address them in clinical practice. The risk-assessment section incorporates suicide-related risk factors documented in the literature and activities tailored to the oncology context [1–8, 12–14].

The final session addresses the collaborative management of identified suicide risk by oncology nurses, patients, families, and the healthcare team. Strategies are based on safety planning interventions [33]—evidence-based risk management practices—and developed using literature that meets the needs of oncology nurses in suicide risk management [12–14]. The session ends with guidance on informing patients, families, and healthcare team members after risk identification, addressing behaviors such as ignoring and stigmatizing suicide, and using approaches that align with nurses’ roles and legal responsibilities. This section also details coping safety strategies, including recognizing situations indicating increased risk, making environmental adjustments, leveraging social support, and identifying emergency contact units to protect cancer patients from suicide in both clinical and hospital settings. The session also emphasizes the importance of educating at-risk patients and their families about the characteristics and significance of psychosocial approaches and psychiatric treatments in managing suicide risk.

**Fig. 1:**
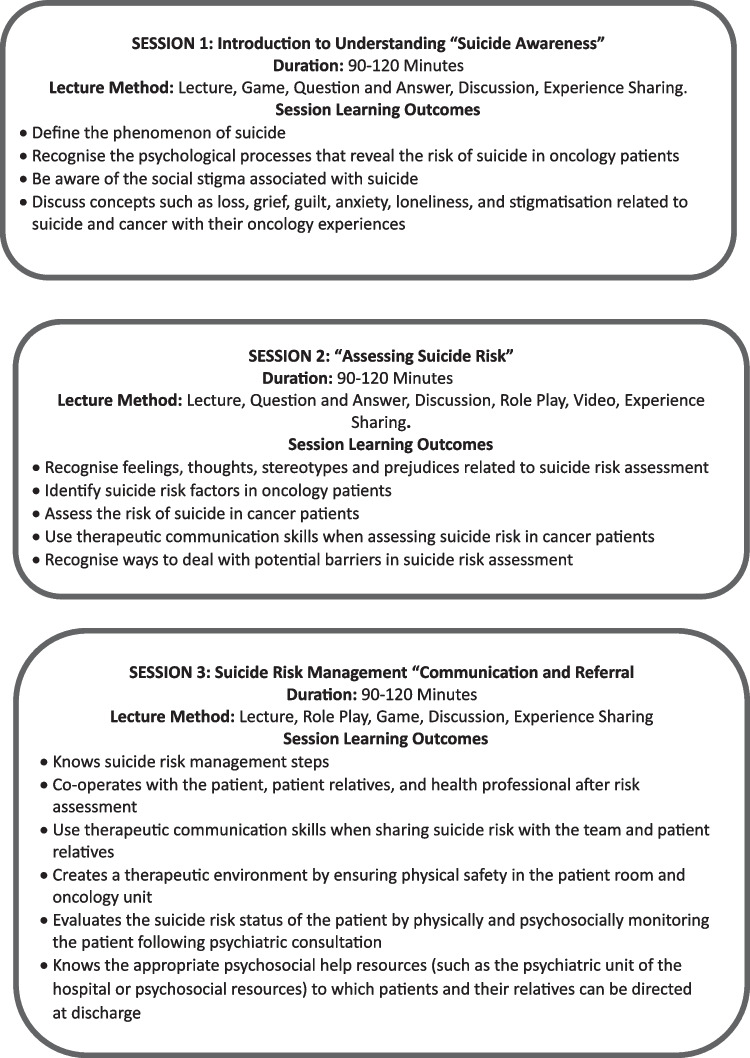
Overview of the OSP sessions

Figure 1 provides an overview of the program sessions and details the learning outcomes of each group session. More detailed session steps for the OSP program are available in the Supplementary Material Section.

### Procedure

After randomizing and allocating the participants, the researchers arranged for the experimental group members to participate in the training sessions at convenient times and days. Subsequently, the researchers instructed the nurses on how to use the Zoom app during the training, and they conducted a pilot trial 1 week before the program started. Then, the researchers distributed the measurement tools to both the experimental and control groups via an online app 1 day prior to the start of the intervention program, and the participants completed the forms. In group programs, limiting the number of members helps them achieve their goals and facilitates group learning, experience sharing, and goal attainment [34]. Thus, the researchers divided the experimental group into four groups: one with nine members, another with ten, and two with twelve participants. OSP sessions were scheduled based on the collective availability of nurses in each group. Nurses were advised that a quiet environment, with suitable locations and times, was essential for effective session delivery. Some nurses arranged suitable settings during working hours, taking into account their workplace context, while others arranged sessions during personal time outside work. The OSP was implemented for these four groups between May 25 and August 2, 2023, on the Zoom online meeting platform led by Researcher 1. At the time of the study, Researcher 1 was a doctoral student in psychiatric nursing with clinical experience in suicide prevention. Researcher 2, a professor of psychiatric nursing with expertise in psychosocial interventions in oncology, supervised all group sessions.

The program sessions were conducted at 5- to 8-day intervals between sessions to ensure the program participants retained and built upon their awareness and knowledge. After the intervention program, participants in the experimental group completed posttests, followed by follow-up tests 3 months later. The researchers matched the control group with the experimental group. They conducted pretests, posttests, and follow-up tests of the control group on the same weeks and days as the experimental group. In addition, the researchers arranged compensation sessions for participants who missed group sessions. In order to maintain group cohesion, only one compensation session was permitted for each participant. Researcher 1 conducted the compensation session by providing the same session that the participant missed, either individually or together with the other group member who missed the same session within the week after the session. Compensation sessions were held for four participants in total. The two participants who were absent from Session 2 received individual compensation sessions. The remaining two participants, who missed the final session, attended a joint compensation session. Participants who did not attend the two sessions of the program were excluded. The follow-up tests were carried out in November 2024.

### Measurements

The primary outcomes of this study were suicide literacy, suicide stigma, and efficacy perception in suicide risk management, and the secondary outcomes were the relationships between changes in these outcomes in the intervention group. Previous studies conducted in the Turkish population found the outcome measures of this study to be valid and reliable [35–37]. The outcome measures are listed below.

#### Literacy of suicide scale (LOSS)

LOSS was developed to assess suicide literacy [38]; the scale, consisting of 27 items, uses a 3-point Likert scale for each item. There are no subscales, only a total scale score ranging from 0 to 27. A higher score on the scale indicates a higher level of literacy about suicide [35].

#### Stigma scale for suicide (SOSS)

SOSS was developed to assess attitudes toward suicide stigma and individuals with a history of suicide attempts [39]. SOSS is a 5-point Likert-type scale that consists of 55 items in total. The scale consists of three subscales: “stigmatization,” “isolation/depression,” and “glorification/normalization.” There is no total score calculation for the scale. Higher scores on the “stigmatization” subscale indicate increased stigmatization toward suicide, higher scores on the “isolation/depression” subscale suggest that individuals attribute suicide to depression and isolation, and higher scores on the “glorification/normalization” subscale show that individuals tend to normalize and glorify suicide [36].

#### Efficacy perception scale for suicide risk management for oncology nurses (EPSSRM)

The EPSSRM was developed to measure oncology nurses’ perceptions of their efficacy in managing suicide risk. The scale consists of 26 items and uses a 5-point Likert scale. The minimum score that can be obtained from this scale is 26, while the maximum is 130, and the scale does not have a specific cut-off point. A higher score indicates that nurses’ perception of the efficacy of their knowledge and skills in managing suicide risk is improving positively [37].

The researchers also prepared a *Descriptive Information Form* to obtain information on the oncology nurses’ age, sex, education level, oncology unit, and working experience.

### Ethical consideration

Before commencing the study, the researchers received ethical permission (Study code: KA21003) from the Clinical Research Ethics Committee of Hacettepe University and obtained institutional permission from two oncology hospitals. Throughout the study, they obtained verbal and written informed consent from all participants. The study followed the Declaration of Helsinki and was registered on ClinicalTrials.gov with the identifier NCT06282263 (first posted on 2024–02–28). Researchers informed the control group, but excluded the experimental group participants, that they would receive the OSP program upon request after completing the study.

### Statistical analysis

The data analysis was conducted using the SPSS 23 statistical program. The chi-square test was used for nominal variables, while the Mann–Whitney *U* test was employed for ordinal variables to determine group differences in pre-intervention descriptive information. Two-way repeated measures analysis of variance (ANOVA) was used to compare the scores of the experimental and control groups for LOSS, SOSS, and EPSSRM. Greenhouse–Geisser correction was used when sphericity was violated. Post hoc analyses and Bonferroni correction were used to compare differences within groups. The effect size of the OSP program was assessed using partial eta squared (*η*^2^). The interpretation of the eta-squared values is that 0.01 represents a small effect, 0.06 a moderate effect, and 0.14 a large effect [22]. The Spearman correlation coefficient evaluated the relationship between the changes in the scale scores. A significance level of *p* < 0.05 is accepted for all statistical tests.

## Results

### Participants

**Fig. 2:**
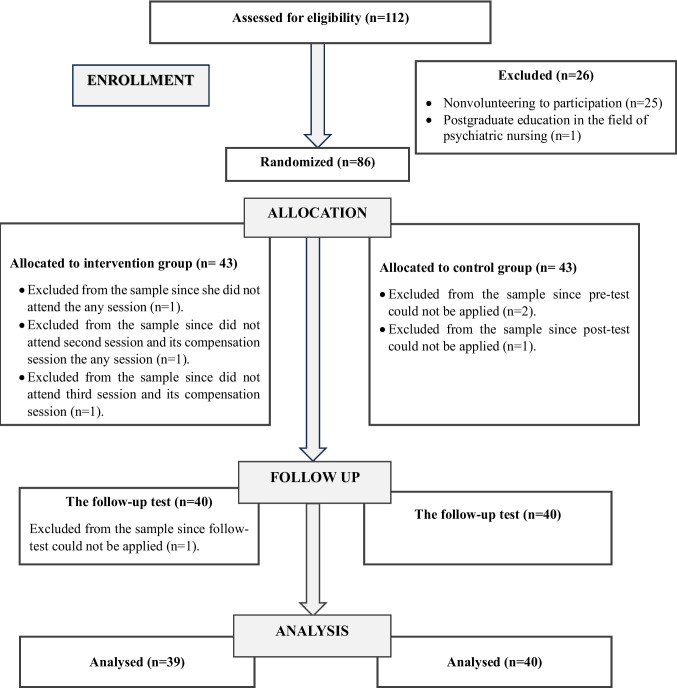
CONSORT flow diagram of the study

In the experimental group, 40 participants completed posttests and 39 completed follow-up tests. In the control group, 40 participants completed posttests and follow-up tests. Figure 2 illustrates the CONSORT flow diagram of the study.

### Descriptive characteristics of oncology nurses

The experimental and control groups are similar in age, duration of experience in the profession, duration of experience in oncology, sex, and working unit. However, the groups differ in education levels. Table 1 provides descriptive characteristics of oncology nurses.

**Table 1:** Descriptive characteristics of oncology nurses

Characteristics	Experimental group		Control group		Statistics	
	*M* [IQR]	Min–Max	*M* [IQR]	Min–Max	Test	*P*
Age	29 [10]	24–48	28 [12]	23–52	*U* = 868.500	0.509
Duration of experience in the profession (month)	60 [126]	12–348	48 [147]	14–300	*U* = 858.000	0.576
Duration of experience in oncology (month)	54 [65]	2–288	45 [78]	3–216	*U* = 857.000	0.583
	***N***	**%**	***N***	**%**		
Sex						
Female	36	90	34	85	*χ*^2^ = 0.457	0.735
Male	4	10	6	15		
Educational Level						
Bachelor’s degree	29	72.5	38	95	*χ*^2^ = 7.440	0.015*
Master’s degree	11	27.5	2	5		
Unit of work in oncology						
Inpatient units	29	72.5	32	80	*χ*^2^ = 0.621	0.599
Outpatient units	11	27.5	8	20		

*U*, Mann–Whitney *U* test; *χ*^2^, Pearson’s chi-square test

*M [IQR]* median [interquartile range], *Min* minimum value, *Max* maximum value

^*^p < 0.05

### Primary outcomes

#### LOSS scores

At the end of the program, the mean LOSS scores significantly increased in the experimental group in the posttest and follow-up tests. ANOVA results show a significant interaction between groups and time with a large effect size (*F* = 47.112; *p* < 0.001; *η*^2^ = 0.380) (Table 2). In the time comparisons within the experimental group, LOSS scores baseline, posttest, and follow-up test scores had significant differences (*F* = 89.531; *p* < 0.001; *η*^2^ = 0.702). Post hoc comparisons showed LOSS posttest (mean differences, MD = 7.13; *p* < 0.001) and follow-up test (MD = 5.51; *p* < 0.001) scores were significantly higher than the baseline scores (Table 3).

**Table 2:** Oncology nurses’ scores of LOSS, SOSS, and EPSSRM

Measure	Experimental group (*N* = 39)			Control group (*N* = 40)			Two-way analysis of variance	
	Mean (SD)			Mean (SD)			Statistics	Group × time
	Baseline	Posttest	3-month follow-up	Baseline	Posttest	3-month follow-up		
LOSS	11.54 (3.42)	18.67 (2.73)	17.05 (3.36)	11.70 (3.12)	11.30 (4.05)	11.45 (4.66)	*F*-value	47.112
							*P*	< 0.001*
							*η*^2^	0.380
SOSS								
Stigmatization	58.54 (18.53)	48.60 (19.11)	49.79 (19.68)	62.80 (21.05)	67.60 (24.83)	64.25 (24.16)	*F*-value	4.471
							*P*	0.013*
							*η*^2^	0.055
Isolation	57.56 (12.93)	53.61 (14.46)	52.36 (14.31)	55.30 (9.33)	56.00 (10.42)	54.90 (11.47)	*F*-value	2.421
							*P*	0.098
							*η*^2^	0.030
Glorification	24.87(6.48)	23.51 (7.94)	24.05 (8.45)	25.33 (6.95)	27.38 (9.50)	25.38 (8.19)	*F*-value	1.937
							*P*	0.148
							*η*^2^	0.025
EPSSRM	80.08 (16.16)	110.79(10.57)	104.90 (12.22)	83.28 (18.72)	88.83 (17.16)	85.35 (20.35)	*F*-value	28.263
							*P*	< 0.001*
							*η*^2^	0.268

*EPSSRM* efficacy perception scale for suicide risk management, *F-value* ANOVA test statistic, *LOSS* literacy of suicide scale, *SD* standard deviation, *SOSS* stigma of suicide scale, *η*^*2*^ partial eta squared

^*^p < *0.05*

**Table 3:** Time comparisons within experimental group

Measure	Experimental group (*N* = 39)			Time comparisons with in experimental group				Statistics	
	Mean (SD)			Mean differences		Mean differences			
	Baseline	Posttest	3-month follow-up	Baseline to posttest within‐group	*p*	Baseline to follow-up within‐group	*p*		
LOSS	11.54 (3.42)	18.67 (2.73)	17.05 (3.36)	7.13	< 0.001*	5.51	< 0.001*	*F*-value	89.531
								*p*	< 0.001*
								*η*^2^	0.702
SOSS									
Stigmatization	58.54 (18.53)	48.60 (19.11)	49.79 (19.68)	9.95	0.024*	8.74	0.032*	*F*-value	6.146
								*p*	0.006*
								*η*^2^	0.144
Isolation	57.56 (12.93)	53.61 (14.46)	52.36 (14.31)	3.95	0.103	5.20	0.034*	*F*-value	5.266
								*p*	0.013*
								*η*^2^	0.122
Glorification	24.87(6.48)	23.51 (7.94)	24.05 (8.45)	1.36	0.769	0.82	1	*F*-value	0.746
								*p*	0.454
								*η*^2^	0.019
EPSSRM	80.08 (16.16)	110.79(10.57)	104.90 (12.22)	30.72	< 0.001*	24.82	< 0.001*	*F*-value	82.346
								*p*	< 0.001*
								*η*^2^	0.684

*EPSSRM* efficacy perception scale for suicide risk management, *F-value* ANOVA test statistic, *LOSS* literacy of suicide scale, *SD* standard deviation, *SOSS* stigma of suicide scale, *η*^*2*^ partial eta squared

^*^p < 0.005

#### SOSS scores

Significant increases were reported in the experimental group’s stigmatization subscale scores from baseline to post-intervention and baseline to follow-up. The results of ANOVA on the stigmatization subscale of SOSS indicate a significant interaction between groups and time with a medium effect size (*F* = 4.471; *p* = 0.013; *η*^2^ = 0.055) (Table 2). The experimental group’s stigmatization subscale baseline, posttest, and follow-up test scores showed significant differences in time comparisons (*F* = 6.146; *p* = 0.006; *η*^2^ = 0.144). Post hoc comparisons showed that the stigmatization posttest (MD = 9.95; *p* = 0.024) and follow-up test (MD = 8.74; *p* = 0.032) scores were significantly higher than the baseline scores (Table 3).

Based on ANOVA results, the experimental and control groups’ SOSS isolation and the SOSS glorification sub-dimension scores did not show a statistically significant difference over time (*p* > 0.05). However, in the time comparisons, the experimental group’s isolation subscale baseline, posttest, and follow-up test scores indicated significant differences in the time comparisons (*F* = 5.266; *p* = 0.013; *η*^2^ = 0.122). Post hoc comparisons show that the follow-up test score for the SOSS isolation sub-dimension is lower than the baseline score (MD = 5.20; *p* = 0.034). The research findings indicated no statistically significant differences between the baseline, posttest, and follow-up test scores of the SOSS glorification subscale in the experimental group (*p* > 0.05) (Table 3).

#### EPSSRM scores

ANOVA results showed that the experimental group’s score changes over time differed from that of the control group. The experimental group’s scores in the EPSSRM posttest and follow-up test increased with a large effect size, while the control group’s scores did not change over time (*F* = 28.263; *p* < 0.001; *η*^2^ = 0.268) (Table 2). In the time comparisons, there were significant differences in baseline, posttest, and follow-up scores, which indicated a difference in the experimental group (*F* = 82.346; *p* < 0.001; *η*^2^ = 0.684). Post hoc comparisons showed that EPSSRM posttest (MD = 30.72; *p* < 0.001) and follow-up test (MD = 24.82; *p* < 0.001) scores were higher than the baseline scores (Table 3).

### Secondary outcomes

The study found a significant positive correlation at a moderate level between LOSS and EPSSRM (*r* = 0.392, *p* = 0.012) in the changes in baseline-posttest scores. Additionally, in the score changes in baseline-follow-up, a low-level negative correlation between LOSS and stigmatization (*r* = −0.385, *p* = 0.016) and a moderate-level negative correlation between LOSS and glorification (*r* = −0.455, *p* = 0.004) were reported (Table 4).

**Table 4:** The correlations between changes in the scales scores in experimental group

		Baseline to posttest					Baseline to follow-up test				
		LOSS	SOSS			EPSSRM	LOSS	SOSS			EPSSRM
			Stigmatization	Isolation	Glorification			Stigmatization	Isolation	Glorification	
LOSS	*r*					392		−385		−455	
SOSS	*p*					0.012*		0.016		0.004*	
Stigmatization	*r*										
	*p*										
Isolation	*r*				419						
	*p*				0.007*						
Glorification	*r*		595								
	*p*		0.001*								
EPSSRM	*r*										
	*p*										

*EPSSRM* efficacy perception scale for suicide risk management, *LOSS* literacy of suicide scale, *SOSS* stigma of suicide scale, *r* Spearman’s correlation coefficient

^*^p < 0.005

## Discussion

The results of this study indicate that the OSP significantly enhanced oncology nurses’ suicide literacy, increased their perceived efficacy in suicide risk management, and reduced suicide stigma. These effects were demonstrated by the experimental group, which exhibited higher suicide literacy at both program completion and follow-up compared to baseline and control groups. The program content specifically addressed oncology-related suicide prevention needs [12–14], allowing nurses to integrate new knowledge with their existing expertise. This integration likely contributed to the observed improvements in suicide literacy. Groups can develop attitudes and behaviors by promoting engagement and supporting members in achieving shared objectives [40]. This process occurs as members learn from one another’s experiences and through role modeling, which enhances motivation. In particular, group-based learning methods such as games, role-play, and experience sharing may have directly contributed to the observed increase in perceived efficacy at program completion and follow-up.

Reducing stigma is essential for effective suicide prevention [41], as positive attitudes facilitate preventive behaviors [42]. In this context, the study found that the program measurably improved nurse attitudes for at least 3 months, indicating a sustained effect. This improvement likely reflects the OSP’s ability to address social and cultural factors. Nevertheless, the program produced only a modest reduction in isolation-related stigma, which may persist because of enduring myths about suicide and the link with social isolation [30].

The findings indicate that increased literacy is moderately associated with perceived efficacy; however, efficacy is also influenced by factors such as attitude and behavior [42, 43], suggesting that changes in literacy may only partially account for variations in efficacy. Moderate positive relationships between glorification and isolation, as well as between glorification and stigmatization, were assessed through pre- and post-program score changes. In contrast, Batterham et al. [39] found that stronger attributions of stigma and isolation were strongly associated with reduced glorification of suicide. Furthermore, the observed negative correlation between suicide literacy and stigmatization is consistent with anticipated outcomes regarding changes in pre-program and follow-up scores.

Finally, our study found that the control and experimental groups had different education levels. Research suggests that nurses’ stigmatization toward suicide reduces with work experience [23, 24], and working in a psychiatric clinic affects nurses’ suicide literacy and stigma [24, 44]. However, the impact of nurses’ education levels on suicide literacy and suicide stigma needs to be clarified. A study conducted in Turkey reported that there was no difference in suicide literacy between nurses with postgraduate education and nurses with undergraduate education [44]. Therefore, the education level disparity between the groups is unlikely to affect our study results.

### Strengths and limitations

The OSP represents a significant contribution to the literature, as it is the first suicide prevention program evaluated in a randomized study involving oncology nurses. Key strengths of this study include its targeted focus on oncology nurses, the comprehensive program design incorporating oncology-specific suicide prevention strategies, and the assessment of outcomes at a 3-month follow-up. A major limitation is the absence of an active placebo control group. Implementing a placebo intervention was considered ethically inappropriate given the critical importance of suicide prevention [45]. Furthermore, including a placebo control group in behavioral research is often impractical due to the unique characteristics of the subject matter [46]. Institutional policies and Ministry of Health standards mandated that all participants continue their scheduled in-service training. Therefore, the effectiveness of the OSP was compared with standard professional development that reflects real-world clinical practice, rather than a no-training condition. Additional limitations include the program’s exclusive online delivery and the inability to blind both participants and researchers.

## Conclusion

The findings of this randomized study indicate that the OSP is associated with increased suicide literacy, enhanced perceived efficacy, and reduced suicide stigma among oncology nurses at both the immediate post-intervention and 3-month follow-up assessments. Although the randomized design reduces potential bias, the absence of an active placebo control suggests that these results should be interpreted as preliminary evidence of the program’s practicality and acceptability. The OSP demonstrates potential to improve competencies in suicide risk management. Future multicenter trials are necessary to evaluate the program’s comparative effectiveness against other active training modules, thereby strengthening the evidence base and validating these findings prior to broader implementation in national prevention strategies. Furthermore, ongoing evaluation of the program’s effectiveness at 6-month and 1-year intervals is essential to ensure sustained impact. Given that the program has been delivered online, conducting and comparing face-to-face implementations is recommended. Such comparisons may yield valuable insights into the program’s effectiveness and further enhance confidence in its efficacy.

## Supplementary Information

Below is the link to the electronic supplementary material.

ESM 1 (DOCX 18.5 KB)

## Data Availability

The data is available from the authors upon reasonable request.

## Declarations

### Reporting method

Consolidated Standards of Reporting Trials (CONSORT).
