# Supplementary material for: The effect of oncology-specific suicide prevention program on oncology nurses’ levels of suicide literacy, suicide stigma, and efficacy perception for suicide risk management: a randomized controlled study
Source: Support Care Cancer. 2026 Jun 8;34(7):626. doi: 10.1007/s00520-026-10854-0 (PMC13246819; doi:10.1007/s00520-026-10854-0)
Supplement: Supplementary file 1 — (DOCX 18.5 KB) [file 520_2026_10854_MOESM1_ESM.docx]

**SUPPLEMENTARY MATERIAL**

**OSP** **SESSION DETAILS**

| **SESSIONS** | **STEPS** |
| --- | --- |
| **SESSION 1:**  **INTRODUCTION TO UNDERSTANDING SUICIDE '' AWARENESS ''** | 1. First, group members were directed to a warm-up activity for an engaging and productive group environment. 2. After the warm-up, group members were informed about the session's purpose, structure and rules and questions were answered. 3. Group members were asked how they feel before starting the program content. 4. Group members were asked to draw and describe the first three scenes that come to mind when the word "suicide" was used. After this instruction, how group members perceived the connotations of "suicide" was discussed. 5. Group members were encouraged to voluntarily share their experiences related to the phenomenon of suicide in order to create a supportive environment and mutual empathy. 6. The results of both qualitative and quantitative research on the phenomenon of suicide in cancer were presented. The similarities and differences between this information and the knowledge and experiences of the group members were discussed. 7. Examples from newspaper news about suicide were given. These news were discussed in the context of suicide awareness and suicide stigma. 8. Group members were informed about the relationship between suicide risk and the distress that a person with cancer may experience, such as loss, grief, loneliness, anxiety, pain, and guilt. After this information, group members were asked to review and discuss their own patients' experiences in the context of this information. 9. At the end of this session, group members were asked what they would like to change with the information provided in the session. 10. The session was summarized, ensuring that all important points are clear and that participants are reassured. |
| **SESSION 2: ASSESSING SUICIDE RISK** | 1. Group members were invited to share their feelings and thoughts since the previous session. The topics discussed in the previous session were summarized. Expectations from the new agenda were discussed. 2. The general principles of suicide risk assessment, when to do it, risk assessment steps, and guidelines were presented. 3. Sample cases were presented. Participants were asked to evaluate suicide risk factors based on the case. 4. Then, information was given about the situations that need to be evaluated urgently in risk assessment. 5. Group members were asked what they think and feel about questioning the suicidal thoughts, intentions and actions of a cancer patient. 6. Before moving on to the stage of assessing the content of suicidal thoughts, intentions and actions, group members role-played games on how to do this. In this stage, one of the group members was asked to be the patient, and the other is the nurse. The patient role asked the nurse role, "If I do not take these medications, how long will it take for me to die, nurse?" Then, the individual's response in the nurse role was awaited. 7. After the role-play, group members were encouraged to share their thoughts and feelings about their roles. 8. Group members' stigma and unrealistic beliefs about talking to the patient about suicide were handled. It was emphasized that talking about suicide was one of the most critical steps in preventing suicide. 9. Group members were informed about how to evaluate patients' suicidal thoughts, intentions, and attempts. 10. Group members were given examples of assessing patients' support resources, safety, and coping mechanisms. 11. A sample video of a suicide risk assessment was shown to the group members. 12. After this video, the nurses were asked to write down their facilitators and barriers to assessing suicide risk on a piece of paper. The nurses' barriers and solutions to these barriers were discussed, and the group members' solution suggestions were integrated with risk management strategies. 13. A sample case was given. Group members were asked to demonstrate the risk assessment steps through role-play, using the information in the sample case, and talk about the risk of suicide with the patient in the case. After the role-play, the group members' role feedback was discussed. 14. The session was summarized, ensuring that all important points are clear and that the participants feel secure. |
| **SESSION 3: SUICIDE RISK MANAGEMENT ''COMMUNICATION AND REFERRAL''** | 1. The topics discussed in the previous session have been summarized. The steps for managing suicide risk were explained to the group members, and a discussion took place on what to do if the patient is deemed to be at risk of suicide as a result of the suicide risk assessment. 2. Collaborative approaches for working with patients, families, and healthcare professionals were discussed with group members. 3. Afterwards, group members were asked to share their feelings and thoughts about discussing the patient's suicide risk with the family and team. They were encouraged to acknowledge both positive and negative emotions regarding this topic. The potential impact of negative feelings on patient care was also discussed. Additionally, the group discussed principles, obstacles, and suggestions for sharing the suicide risk with the family and team. 4. Group members were asked how to share the suicide risk with the patient's relatives. Information is provided about the approaches the nurse can adopt against the possible adverse reactions of the patient's relatives. 5. What can be planned in the physical environment to prevent suicide attempts was explained. 6. In cases where a psychiatric consultation was not requested for a patient at risk of suicide, but the nurse thinks the risk is still present, what can be done was asked. In this case, the group members were informed about what nurses can do. 7. The care of patients using psychiatric medications was being discussed with the group members. Myths about psychiatric medications were addressed, and information was provided about the basic principles of psychiatric medication use. 8. Information that could be given to the patient and their relatives regarding preventing suicide risk during discharge was discussed with the group members. The importance of patients at risk of suicide taking their medications regularly and developing post-discharge strategies to manage suicide risk (for example, seeking help from family or relatives as soon as possible when suicidal thoughts occur) was emphasized. 9. During discharge training in oncology clinics, the group members were reminded of the importance of referring patients to appropriate mental health services. 10. The last session was summarized. The group leader clarifies unclear issues. 11. Group members were asked their feelings and thoughts about the program. 12. Group members were given contact numbers to reach the researchers. |
